# Involvement of the Carboxyl-Terminal Region of the Yeast Peroxisomal Half ABC Transporter Pxa2p in Its Interaction with Pxa1p and in Transporter Function

**DOI:** 10.1371/journal.pone.0104892

**Published:** 2014-08-13

**Authors:** Cheng-Yi Chuang, Ling-Yun Chen, Ru-Huei Fu, Shih-Ming Chen, Ming-Hua Ho, Jie-Mau Huang, Chia-Chi Hsu, Chien-Cheng Wang, Meng-Shian Chen, Rong-Tzong Tsai

**Affiliations:** 1 Institute of Biochemistry and Biotechnology, College of Medicine, Chung Shan Medical University, Taichung, Taiwan; 2 Department of Biochemistry, School of Medicine, Chung Shan Medical University, Taichung, Taiwan; 3 Graduate Institute of Immunology, China Medical University, Taichung, Taiwan; College of Medicine, University of South Florida, United States of America

## Abstract

**Background:**

The peroxisome is a single membrane-bound organelle in eukaryotic cells involved in lipid metabolism, including β-oxidation of fatty acids. The human genetic disorder X-linked adrenoleukodystrophy (X-ALD) is caused by mutations in the *ABCD1* gene (encoding ALDP, a peroxisomal half ATP-binding cassette [ABC] transporter). This disease is characterized by defective peroxisomal β-oxidation and a large accumulation of very long-chain fatty acids in brain white matter, adrenal cortex, and testis. ALDP forms a homodimer proposed to be the functional transporter, whereas the peroxisomal transporter in yeast is a heterodimer comprising two half ABC transporters, Pxa1p and Pxa2p, both orthologs of human ALDP. While the carboxyl-terminal domain of ALDP is engaged in dimerization, it remains unknown whether the same region is involved in the interaction between Pxa1p and Pxa2p.

**Methods/Principal Findings:**

Using a yeast two-hybrid assay, we found that the carboxyl-terminal region (CT) of Pxa2p, but not of Pxa1p, is required for their interaction. Further analysis indicated that the central part of the CT (designated CT_2_) of Pxa2p was indispensable for its interaction with the carboxyl terminally truncated Pxa1_NBD. An interaction between the CT of Pxa2p and Pxa1_NBD was not detected, but could be identified in the presence of Pxa2_NBD-CT_1_. A single mutation of two conserved residues (aligned with X-ALD-associated mutations at the same positions in ALDP) in the CT_2_ of the Pxa2_NBD-CT protein impaired its interaction with Pxa1_NBD or Pxa1_NBD-CT, resulting in a mutant protein that exhibited a proteinase K digestion profile different from that of the wild-type protein. Functional analysis of these mutant proteins on oleate plates indicated that they were defective in transporter function.

**Conclusions/Significance:**

The CT of Pxa2p is involved in its interaction with Pxa1p and in transporter function. This concept may be applied to human ALDP studies, helping to establish the pathological mechanism for CT-related X-ALD disease.

## Introduction

ATP-binding cassette (ABC) transporters belong to a superfamily of membrane proteins that transport a range of bioactive molecules across the cellular membrane. In general, functional ABC transporters comprise four domains: two transmembrane domains (TMDs) involved in substrate binding and translocation, and two nucleotide-binding domains (NBDs) involved in ATP binding and hydrolysis [1]. These ABC transporter domains can be located in a single polypeptide (such as Arabidopsis Comatose and cystic fibrosis transmembrane conductance regulator [CFTR]), in two polypeptides (such as human ALDP, the human transporters associated with antigen processing TAP1 and TAP2, and the yeast peroxisomal transporters Pxa1p and Pxa2p), or in four polypeptides (such as the *Escherichia coli* maltose transporter) [2]–[10]. In addition to the common ABC transporters TMD and NBD, some possess an extra sequence or accessory domain to regulate ATPase activity, channel opening, and interactions between NBD and NBD or NBD and TMD, as in the case of SUR2x/Kir6.2, maltose transporter MalFGK_2_, molybdate/tungstate transporter ModBC, human CFTR, and DrrA [11]–[15].

**Figure 1:**
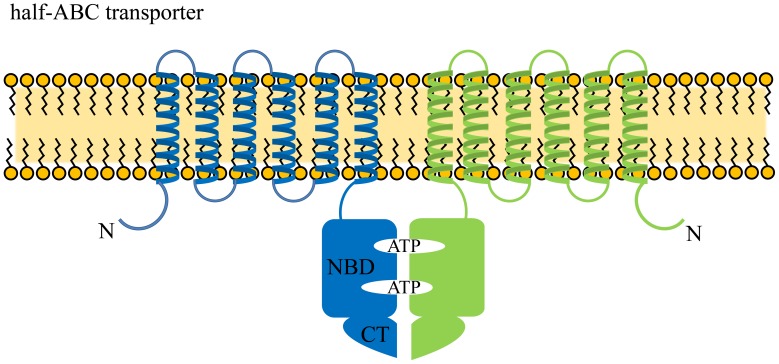
General schematic of the functional ABC transporter consisting of two half ABC transporters. In general, each half ABC transporter contains a transmembrane domain (TMD) and a nucleotide-binding domain (NBD) with or without the carboxyl-terminal region (CT).

Peroxisome, a single membrane organelle in the eukaryotic cell, is involved in lipid metabolism, including β-oxidation of fatty acids. The peroxisome membrane is equipped with peroxisomal ABC transporters for transferring long-chain or extremely long-chain fatty acids from the cytosol into the peroxisome [16]. In humans, the importance of the peroxisomal ABC transporter is demonstrated by the common genetic disorder, X-linked adrenoleukodystrophy (X-ALD), caused by mutations in the *ABCD1* gene (encoding ALDP, a peroxisomal half ABC transporter). The disease is characterized by defective peroxisomal β-oxidation and accumulation of very long-chain fatty acids in brain white matter, adrenal cortex, and testis, resulting in cerebral demyelination, nerve degeneration, and adrenocortical and testicular insufficiency [17]–[20]. Besides ALDP, there exist two other half ABC transporters, ALDRP and PMP70 [21]–[23]. They both share amino acid sequence homology with ALDP. ALDP has been shown to form a homodimer with itself or a heterodimer with ALDRP or PMP70 through the interaction between their carboxyl-terminal regions (CTs) [4], [24] (Fig. 1). Overexpressed human ALDP can functionally complement the yeast strain deleted in the peroxisomal ABC transporters Pxa1p and Pxa2p, as shown by the restoration of cell growth on oleate medium and by β-oxidation activity, suggesting that the homodimer of ALDP is the functional transporter in vivo [25]. In addition, murine ALDP, ALDRP, and PMP70 were shown to be regulated differentially during mouse brain development, indicating that these proteins may not function together at the same time and that they form the functional homodimer at different developmental stages [26]. One report, however, suggests that overexpressed ALDRP exhibits substrate specificity overlap and interaction with ALDP in a human cell model, indicating the existence of a functional heterodimer comprising ALDP and ALDRP [27].

In *Saccharomyces cerevisiae*, there are two half ABC transporters in the peroxisome, Pxa1p and Pxa2p, both of which are orthologs of human ALDP [7], [28]. Co-immunoprecipitation of Pxa1p and Pxa2p has suggested physical interaction between them [9]. Genetic analysis results also indicated that both Pxa1p and Pxa2p are functionally indispensable [8], [29]. The single-gene deletion strains *Δpxa1* or *Δpxa2* exhibited the same phenotype (inability to grow on an oleic acid plate and impaired β-oxidation activity) as the double-gene deletion strain *Δpxa1/pxa2*. Although the Pxa1p and Pxa2p heterodimer has been proposed as the functional transporter, the interaction regions in Pxa1p and Pxa2p have not yet been determined. Like ALDP, both Pxa1p and Pxa2p have a CT, and the significance of this region in protein-protein interaction has not yet been analyzed.

Using a yeast two-hybrid assay in the present study, we found that the CT at the end of NBD of Pxa2p is required for Pxa1p-Pxa2p interaction. To define the interaction region in detail, we made a series of deletions in the CT of Pxa2p and assayed the effect of the mutations on protein-protein interaction. The results showed that the central part of the CT (designated CT_2_) of Pxa2p was most important for the interaction between Pxa1_NBD and Pxa2_NBD. This conclusion was supported by a pull-down assay. The failure to detect an interaction between the CT of Pxa2p and Pxa1_NBD implied that it functioned in the Pxa1p-Pxa2p interaction not through physical contact with Pxa1p, but rather, by indirect means dependent on the existence of Pxa2_NBD-CT_1_. Further analysis revealed that a point mutation of conserved residues in the CT_2_ of Pxa2_NBD-CT abolished its interaction with Pxa1p_NBD or Pxa1p_NBD-CT and displayed proteolysis profiles that differed from that of the wild type. Functional analysis of these mutants indicated that they were defective in transporter function. Taken together, these results suggest that the CT region is required to maintain the native structure of Pxa2_NBD, a prerequisite for the interaction between Pxa1p and Pxa2p and the transporter function.

## Results

### Involvement of the CT of Pxa2p in the interaction between Pxa1_NBD-CT and Pxa2_NBD-CT

**Figure 2:**
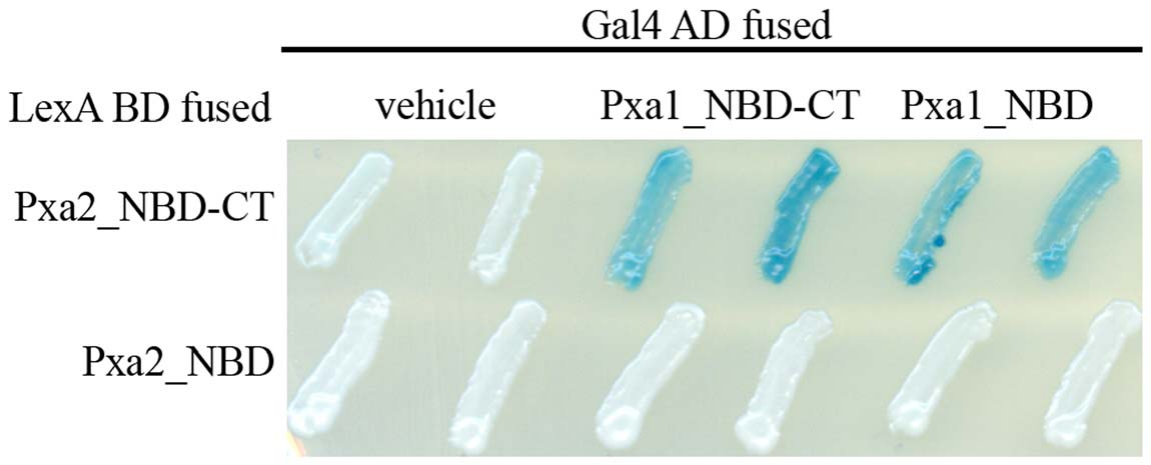
Two-hybrid analysis of interactions between Pxa2_NBD-CT (or Pxa2_NBD) and Pxa1_NBD-CT (or Pxa1_NBD). The EGY48 (pSH18-34) reporter strains that express the indicated hybrid proteins (with the Pxa2 fragment fused to LexA BD and the Pxa1 fragment fused to Gal4 AD) were analyzed for β-galactosidase activity on an X-gal plate. Plates were incubated for 16 h at 30°C. Blue indicates the existence of a protein-protein interaction between hybrid proteins.

The CT of the human homolog ALDP has been shown to be engaged in protein-protein interaction with itself [4]. To investigate the importance of the CT of Pxa1p and Pxa2p in Pxa1p-Pxa2p interaction, we performed a yeast-two hybrid assay. Because inclusion of the TMD may give rise to false negative results as a consequence of protein aggregation or degradation (data not shown), we fused the soluble region of Pxa1p and Pxa2p only, which included an NBD and CT region (Pxa1_NBD-CT and Pxa2_NBD-CT) to the GAL4 activation domain (Gal4 AD) and the LexA DNA-binding domain (LexA BD), respectively. The CT of Pxa1p ranges from residues 775 to 870 and that of Pxa2p ranges from residues 678 to 853. The CT-truncated proteins were also constructed and designated Pxa1_NBD and Pxa2_NBD, respectively. Results from the yeast two-hybrid assay revealed that Pxa2_NBD-CT, but not Pxa2_NBD, interacts with Pxa1_NBD-CT or Pxa1_NBD, suggesting that the CT of Pxa2p was required for Pxa1p-Pxa2p interaction (Fig. 2). The C-terminal deletion mutant of Pxa1p, Pxa1_NBD, preserved the ability to interact with Pxa2_NBD-CT, indicating that the interactions between the NBDs of Pxa1p and Pxa2p could occur in the absence of the CT of Pxa1p. Because the CT of Pxa2p has critical effects on the interactions between the NBDs of these two proteins, we were prompted to identify how the CT of Pxa2p influences the interactions of the two NBDs. To more specifically focus on the role played by the CT of Pxa2p and to simplify the variables in the interactions of the two NBDs, we used the yeast two-hybrid combination Pxa1_NBD and Pxa2_NBD_CT as starting material.

### Effect of the C-terminal deletion of Pxa2_NBD-CT on the interaction between Pxa2_NBD-CT and Pxa1_NBD

**Figure 3:**
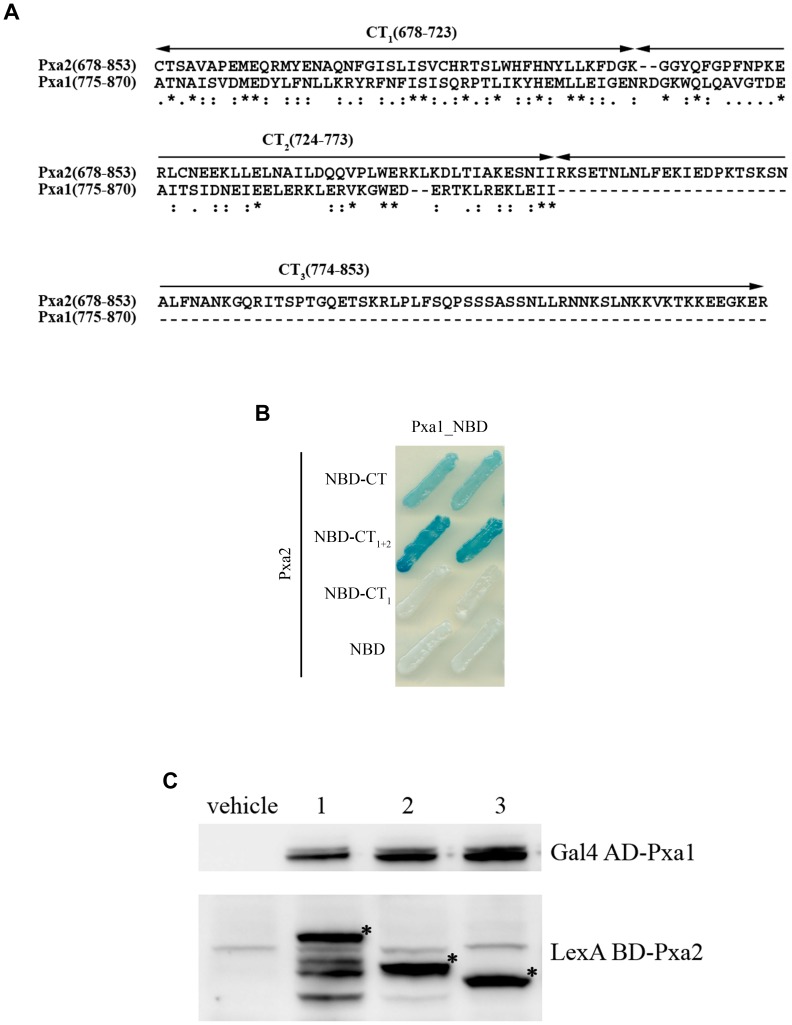
Effect of the C-terminal deletion of Pxa2_NBD-CT on the interaction between Pxa2_NBD-CT and Pxa1_NBD. (A) Multiple sequence alignment of the C-terminal amino acid sequences of the peroxisomal ABC transporter protein Pxa2p (678–853) and Pxa1p (775–870) by the ClustalW2 program. The C-terminal sequences of Pxa1p (775–870) and Pxa2p (678–853) were analyzed with the ClustalW2 program on the EMBL-EBI website (http://www.ebi.ac.uk/Tools/msa/clustalw2/). Pxa2p has an extreme C-terminal region (774–853) that does not exist in Pxa1p. We designated this as the CT_3_ region. The region before CT_3_ was divided into two regions, designated CT_1_ (678–723) and CT_2_ (724–773). * indicates identical residues; indicates conserved residues; indicates semi-conserved residues. (B) Two-hybrid analysis of the interactions between Pxa2_NBD-CT (or its deletion mutants) and Pxa1_NBD. Two-hybrid plasmids were co-transformed into EGY48 (pSH18-34). The yeast transformants were streaked on an X-gal plate and incubated for 16 h at 30°C. Blue indicates the existence of the heterodimeric interaction. (C) Expression levels of the yeast two-hybrid proteins in yeast transformants. The total yeast extracts were prepared by the glass bead method and assayed by Western blot probed with anti-LexA BD and anti-Gal4 AD antibodies. Lane 1: yeast co-expressing LexA BD-Pxa2_NBD-CT and Gal4 AD-Pxa1_NBD; lane 2: yeast co-expressing LexA BD-Pxa2_NBD-CT_1+2_ and Gal4 AD-Pxa1_NBD; lane 3: yeast co-expressing LexA BD-Pxa2_NBD-CT_1_ and Gal4 AD-Pxa1_NBD.

We further mapped the interaction motif within the CT of Pxa2_NBD-CT that was required for its interaction with the CT-truncated Pxa1_NBD. Sequence alignment of the CT regions of Pxa1p and Pxa2p with the ClustalW algorithm (Fig. 3A) revealed an extra sequence at the extremely carboxyl end of Pxa2p (from residue 774 to 853) that is absent in Pxa1p. Other regions of the CT exhibited about 21% identity (data not shown). From sequence comparison, we established that the CT of Pxa2p was divided into three parts: CT_1_ (from residue 678 to 723), CT_2_ (from residue 724 to 773), and CT_3_ (from residue 774 to 853) (Fig. 3A). To determine which part of the CT of Pxa2p is important in protein-protein interaction with Pxa1_NBD, we analyzed the effect of various CT deletions of Pxa2_NBD-CT on protein-protein interaction by using a yeast two-hybrid assay. To our surprise, deletion of CT_3_ did not attenuate the interaction between Pxa1_NBD and Pxa2_NBD-CT. Instead, this deletion slightly enhanced their interaction. Simultaneous deletion of CT_2_ and CT_3_, however, completely made this interaction undetectable (Fig. 3B). Western blot results indicated that all of the mutant proteins were stable (Fig. 3C). From these results, we concluded that the CT_2_ of Pxa2_NBD-CT was essential for its interaction with Pxa1_NBD.

### Analysis of co-purification of the CT-deleted mutant proteins of Pxa2_NBD-CT with Pxa1_NBD

**Figure 4:**
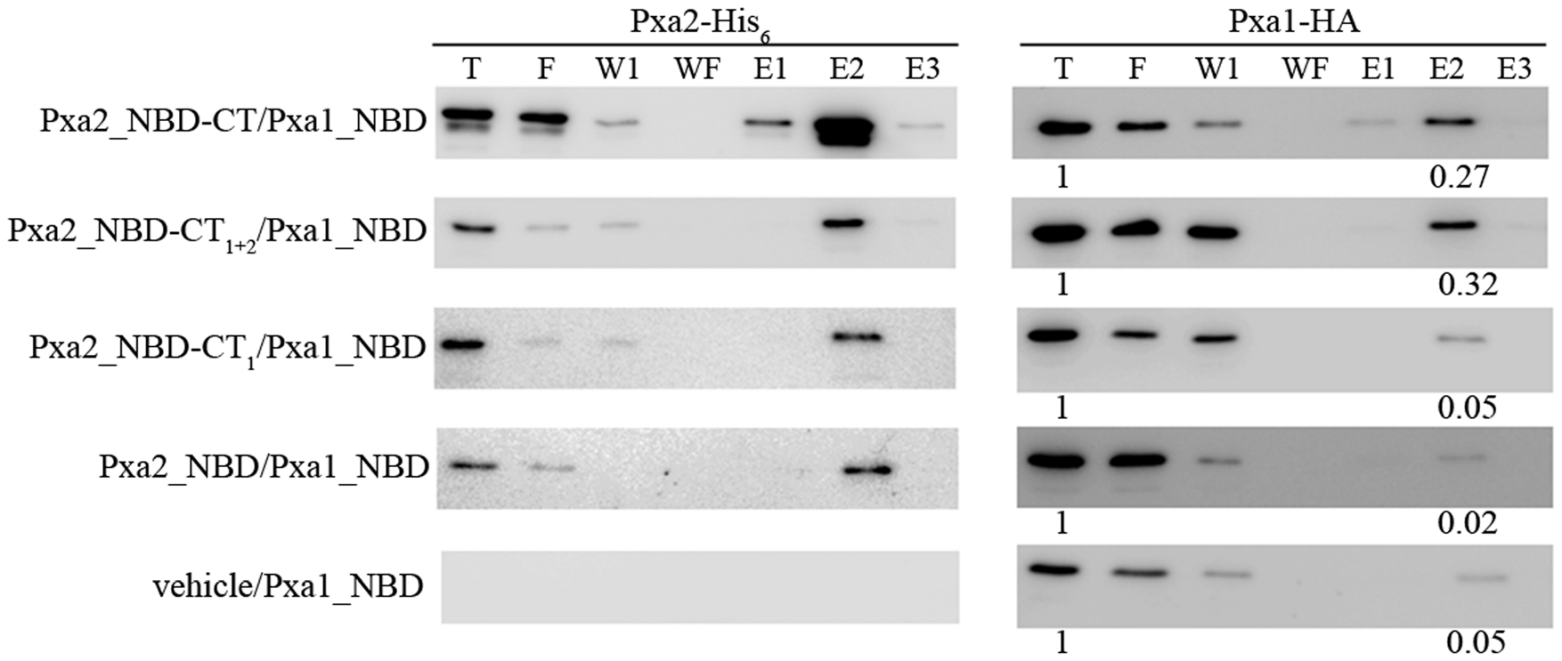
Analysis of co-purification of the CT-deleted mutant proteins of Pxa2_NBD-CT with Pxa1_NBD. The various CT-deleted proteins of Pxa2_NBD-CT-His_6_ were co-expressed with Pxa1_NBD-HA in yeast strain BJ2168, and yeast cells were disrupted by sonication. The total yeast extracts were subjected to a pull-down assay using Ni^2+^-NTA chromatography. The presence of Pxa2-His_6_ or Pxa1-HA in the total extract (T), flow-through (F), wash-one (W1), wash-final (WF), elute-one (E1), elute-two (E2), and elute-three (E3) fractions was revealed by Western blot probed with anti-HA antibody for Pxa1-HA (right panel) and anti-His antibody for Pxa2-His6 (left panel). Protein intensity was analyzed by using GeneTools software (Syngene). Protein intensity of the E2 fraction was normalized to that of the T fraction for each experiment.

To confirm that the CT_2_ of Pxa2_NBD-CT was required for the Pxa1_NBD/Pxa2_NBD-CT interaction, we performed a pull-down assay by fusing the His tag to the CT of Pxa2_NBD-CT and its deleted derivatives and the V5 tag to the CT of the Pxa1_NBD. The fusion proteins were constructed in the yeast expression vector pRS414-Gal or pRS416-Gal, and their transcription was driven by the *Gal* promoter. Full-length Pxa2_NBD-CT, or its deleted derivatives, was co-expressed with Pxa1_NBD in yeast strain BJ2168. The whole cell extracts were passed through a Ni-NTA column. By comparing the E2 fractions, we found that the amount of Pxa1_NBD co-purifying with Pxa2_NBD-CT_1+2_ was comparable to that co-purifying with Pxa2_NBD-CT (Fig. 4). In contrast, the amounts of Pxa1_NBD co-purifying with Pxa2_NBD-CT_1_ or Pxa2_NBD were similar to that co-purifying with the empty vector, indicating decreased binding affinity with Pxa1_NBD in the absence of CT_2_. These results suggested that the CT_2_ of Pxa2_NBD-CT was required for efficient interaction between Pxa2_NBD-CT and Pxa1_NBD, consistent with the yeast two-hybrid results.

### The CT of Pxa2_NBD-CT (Pxa2_CT) promotes its interaction with Pxa1_NBD in an indirect and Pxa2_NBD-CT_1_-dependent manner

**Figure 5:**
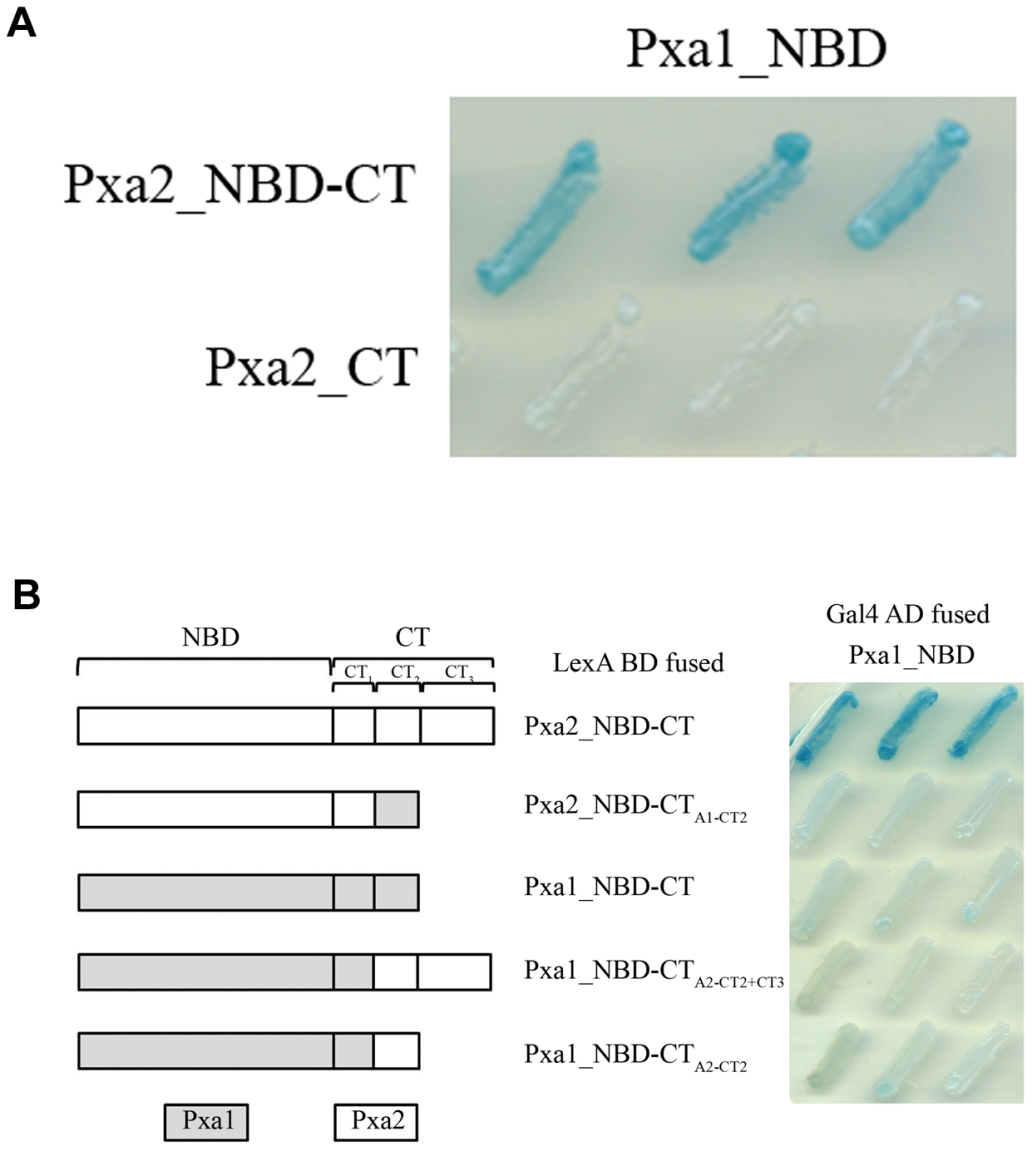
The CT of Pxa2_NBD-CT (Pxa2_CT) promotes its interaction with Pxa1_NBD in an indirect and Pxa2_NBD-CT_1_-dependent manner. (A) Two-hybrid analysis of the interaction between the CT of Pxa2_NBD-CT (Pxa2_CT) and Pxa1_NBD. Two-hybrid plasmids containing Pxa2_CT and Pxa1_NBD were co-transformed into EGY48 (pSH18-34). The yeast transformants were streaked on an X-gal plate and incubated for 16 h at 30°C. Blue indicates the existence of a protein-protein interaction. (B) The effect of the NBD-CT_1_ substitution in Pxa2_NBD-CT on its interaction with Pxa1_NBD. The yeast two-hybrid plasmid containing Pxa1_NBD-CT or Pxa2_NBD-CT with the substitution by NBD-CT_1_ was co-transformed with the plasmid containing Pxa1_NBD into EGY48 (pSH18-34). The yeast transformants were streaked on an X-gal plate and incubated for 16 h at 30°C. Blue indicates the existence of a protein-protein interaction.

We next addressed whether the interaction between Pxa2_NBD-CT and Pxa1_NBD was due to direct interaction between the CT of Pxa2_NBD-CT (Pxa2_CT in Fig. 5A) and Pxa1_NBD. The yeast two-hybrid assay indicated that the CT of Pxa2_NBD-CT alone did not interact with Pxa1_NBD directly, ruling out direct involvement of this region in protein-protein interaction (Fig. 5A). Moreover, we could not detect an interaction between Pxa2_NBD-CT and Pxa1_NBD, as the NBD-CT_1_ region in Pxa2_NBD-CT was substituted with that of Pxa1p, indicating that a specific interaction existed between the NBD and the CT in Pxa2p (Fig. 5B). These results suggested that the CT of Pxa2_NBD-CT might have an indirect and Pxa2_NBD-CT_1_-dependent function to promote Pxa1_NBD/Pxa2_NBD-CT interaction.

### Effect of CT_2_ point mutations of Pxa2p on the interaction between Pxa2_NBD-CT and Pxa1_NBD

**Figure 6:**
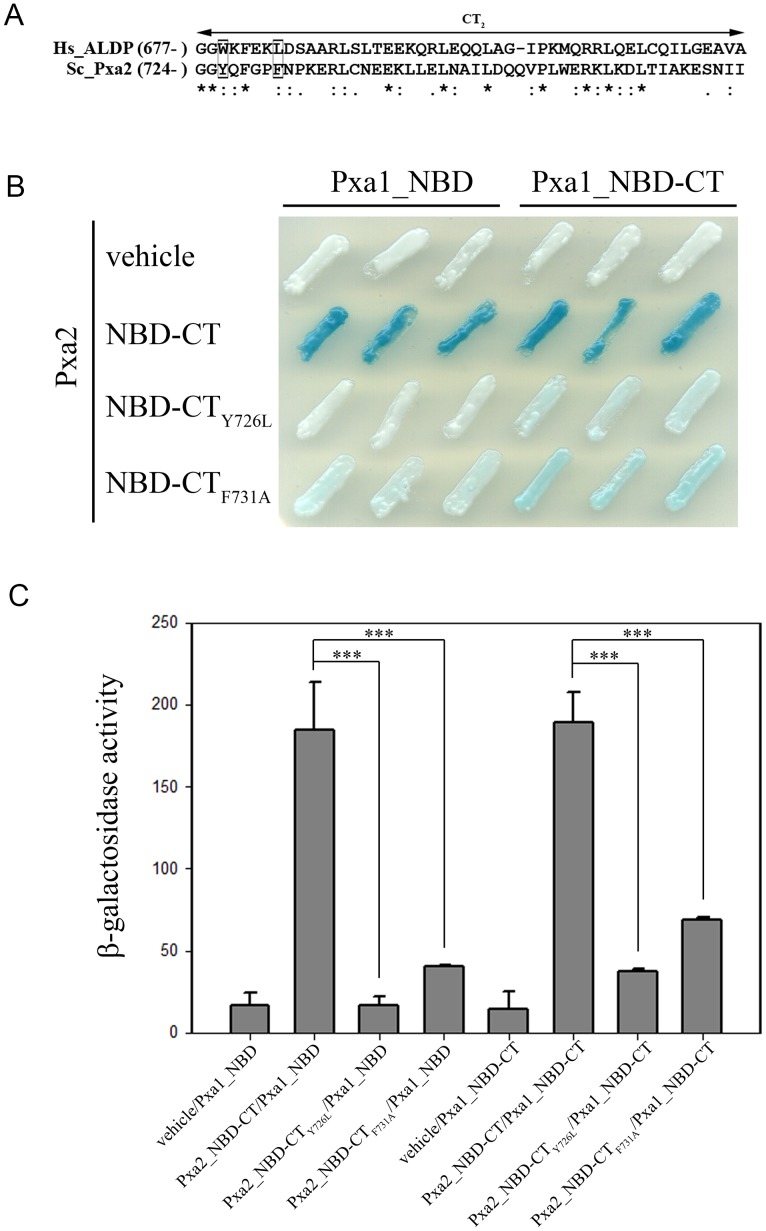
Effect of CT_2_ point mutations of Pxa2p on the interaction between Pxa2_NBD-CT and Pxa1_NBD. (A) The sequence alignment between the CT_2_ sequences of ALDP (677–725) and Pxa2p (724–773) was performed by the ClustalW2 program on the EMBL-EBI website. The conserved amino acids (Y726 and F731) in the CT_2_ of Pxa2p corresponded to the point mutations W679R and L684P in ALD. (B) The mutations Y726L and F731A were individually introduced into Pxa2_NBD-CT to construct yeast two-hybrid plasmids containing Pxa2_NBD-CT_Y726L_ and Pxa2_NBD-CT_F731A_. The yeast two-hybrid analysis of Pxa2_NBD-CT_Y726L_ and Pxa2_NBD-CT_F731A_ with Pxa1_NBD and Pxa1_NBD_CT was performed as described above. (C) The yeast two-hybrid interactions of Pxa2_NBD-CT_Y726L_ and Pxa2_NBD-CT_F731A_ with Pxa1_NBD and Pxa1_NBD_CT were quantified by using the Yeast β-Galactosidase Assay Kit (Thermo). Data are shown as mean ± SD from three independent experiments (n = 3, ***p<0.001).

To confirm whether the CT of Pxa2_NBD-CT promotes Pxa1_NBD/Pxa2_NBD-CT interaction, we constructed point mutations in the CT_2_ of Pxa2p and analyzed the effects of these mutations on Pxa1_NBD/Pxa2_NBD-CT interaction. Because Pxa2p is the homolog of human ALDP, we searched the human ALD genetic disorder database (http://www.x-ald.nl/) for possible mutation targets. We indeed found ALD disease-related point mutations in the CT_2_ of ALDP: W679R and L684P. We then performed the CT_2_ sequence alignment of Pxa2p and ALDP by using the ClustalW2 algorithm (Fig. 6A). Two amino acid residues in Pxa2p, Y726 and F731, were corresponding to W679 and L684 in ALDP, respectively. We thus constructed the point mutations Y726L and F731A in Pxa2_NBD-CT individually. Protein-protein interactions of Pxa2_NBD-CT_Y726L_ and Pxa2_NBD-CT_F731A_ with Pxa1_NBD and Pxa1_NBD-CT were examined by performing the yeast two-hybrid assay. When combined with Pxa1_NBD-CT or Pxa1_NBD, the colors generated by Pxa2_NBD-CT_Y726L_ and Pxa2_NBD-CT_F731A_ on the X-gal plate were white or white-like, significantly different from the blue generated by wild-type Pxa2_NBD-CT. These results indicated that both mutations in Pxa2_NBD-CT, Y726L and F731A, had a significant impact on the interaction between Pxa2_NBD-CT and Pxa1_NBD or Pxa2_NBD-CT and Pxa1_NBD-CT (Fig. 6B). We also used the Yeast β-Galactosidase Assay Kit (Thermo) to more exactly quantify the extent to which the interactions were affected by these point mutations. The results showed that the activities generated by Pxa2_NBD-CT_Y726L_/Pxa1_NBD and Pxa2_NBD-CT_F731A_/Pxa1_NBD were decreased dramatically as compared with that of Pxa2_NBD-CT/Pxa1_NBD. Moreover, the activities generated by Pxa2_NBD-CT_Y726L_/Pxa1_NBD-CT and Pxa2_NBD-CT_F731A_/Pxa1_NBD-CT were decreased significantly as compared with that of Pxa2_NBD-CT/Pxa1_NBD-CT (Fig. 6C). These results suggested that the disruption in the interaction between Pxa1p and Pxa2p by the mutations in the CT_2_ of Pxa2p was statistically significant, consistent with previous yeast two-hybrid data (Fig. 6B). In addition, the mutation effects on the interaction of Pxa2_NBD-CT with Pxa1_NBD and Pxa2_NBD-CT with Pxa1_NBD-CT were similar, indicating that the deletion of the CT of Pxa1p had no obvious effect on protein-protein interaction.

### Limited proteolysis analysis of Pxa2_NBD-CT_Y726L_ and Pxa2_NBD-CT_F731A_ by proteinase K

**Figure 7:**
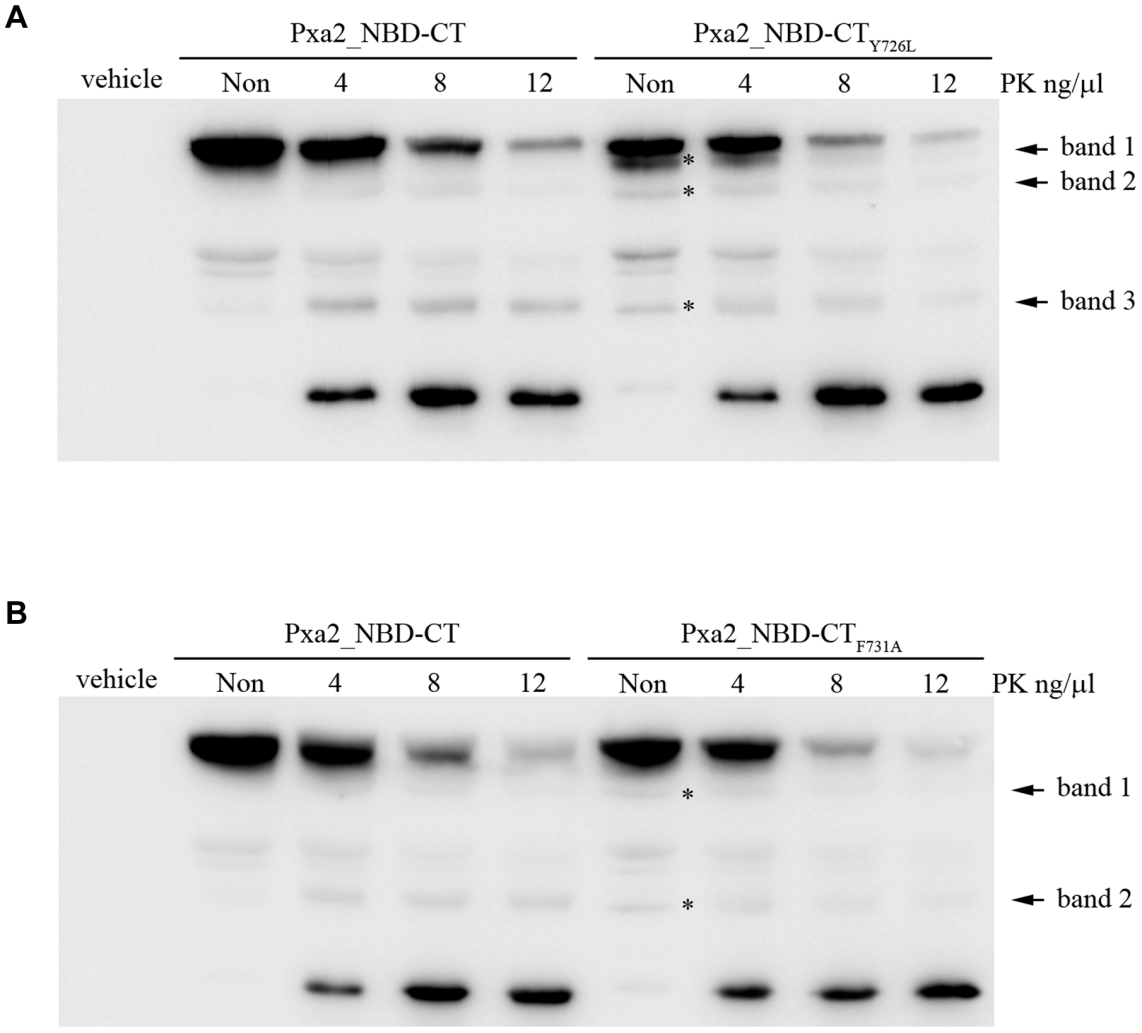
Limited proteolysis analysis of Pxa2_NBD-CT_Y726L_ and Pxa2_NBD-CT_F731A_ by proteinase K. (A) Equal aliquots of total yeast extracts containing Pxa2_NBD-CT_Y726L_ were placed into each tube. Proteinase K was added to each tube with a serial dilution of 0, 4, 8, and 12 µg/ml. After treatment, proteins were analyzed by SDS-PAGE and Western blot probed with anti-His-tag antibodies. The vehicle represented the vector-only control. The different peptide bands between wild type and mutant were marked with an asterisk and labeled band 1, band 2, and band 3. (B) The experimental procedure for limited proteolysis of Pxa2_NBD-CT_F731A_ was the same as in A. The different peptide bands between wild type and mutant were labeled band 1 and band 2.

Since the CT of Pxa2_NBD-CT has an indirect and Pxa2_NBD-CT_1_-dependent role in promoting Pxa1_NBD/Pxa2_NBD-CT interaction, we speculate that the CT of Pxa2p could interact specifically with its NBD to form an interaction-competent structure for interacting with Pxa1_NBD. To explore this possibility, we examined the structural properties of Pxa2_NBD-CT (control), Pxa2_NBD-CT_Y726L_, and Pxa2_NBD-CT_F731A_ by performing a limited proteolysis assay. The samples were prepared by digesting Pxa2_NBD-CT protein using serial dilutions of proteinase K. As compared with the digestion profiles of Pxa2_NBD-CT and Pxa2_NBD-CT_Y726L_, there appeared to be three extra bands in the mutant protein in the untreated sample and in the sample treated with 4 ng/µl proteinase K (Fig. 7A, bands 1, 2, and 3). Less difference was observed between the digestion profile of Pxa2_NBD-CT and that of Pxa2_NBD-CT_F731A_. There appeared to be two extra bands in the mutant protein in the untreated sample (Fig. 7B, bands 1 and 2). These results are consistent with the proposition that the point mutations in the CT_2_ of Pxa2p may have altered the Pxa2_NBD structure, thus diminishing the interaction between Pxa2_NBD-CT and Pxa1_NBD.

### Functional analysis of Pxa2_NBD-CT_Y726L_ and Pxa2_NBD-CT_F731A_ by oleate plate assay

**Figure 8:**
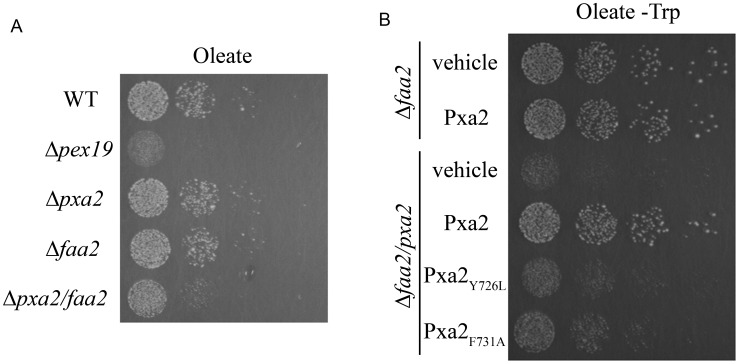
Functional analysis of Pxa2_NBD-CT_Y726L_ and Pxa2_NBD-CT_F731A_ by oleate plate assay. (A) A five-fold dilution series of the yeast strains deleted in *pex19*, *pxa2*, *faa2*, and *pxa2/faa2* were dropped onto the surface of an oleate plate, followed by incubation at 30°C for 5-7 days. (B) The plasmid expressing *PXA2* or the mutant gene was delivered into the *Δfaa2* or *Δfaa2/Δpxa2* strain. Functional analysis of Pxa2_NBD-CT_Y726L_ and Pxa2_NBD-CT_F731A_ was determined using the same procedure as in A. For plasmid selection, the oleate plate used in B lacked tryptophan (Trp).

To identify the functional importance of Y726 and F731 in the CT_2_ of Pxa2p *in vivo*, we use an oleate plate assay to examine its ABC transporter function for transporting oleic acid from the cytosol to the peroxisome. To date, two pathways have been discovered for transporting oleic acid (a long-chain fatty acid) from the cytosol to the peroxisome in yeast. One is performed by the transporter comprising Pxa1p and Pxa2p, and the other is mediated by Faa2p (a peroxisomal acyl-CoA synthetase) [30], [31]. The single-gene knock-out mutant *pxa1*, *pxa2*, or *faa2* preserves partial ability to transport oleic acid, but the double knock-out mutant *pxa1/faa2* or *pxa2/faa2* loses most of its transport function for oleic acid [8]. In addition, peroxisomes are the only organelles to digest oleic acid in yeast, and yeast cells cannot survive in the oleate plate (using oleic acid as a sole carbon source) when these two pathways are shut down [30]. To simplify the functional assay and focus on Pxa2p function, we needed to preserve the Pxa1p-Pxa2p pathway and abolish the Faa2p pathway. We constructed a double knock-out strain (*Δfaa2/pxa2*) as the two-pathway-off control, single-gene knock-out strains (*Δpxa2 and Δfaa2*) as single-pathway-off controls, and an *Δpex19* strain as a null-peroxisome control [32]. The growth rate of the *Δpxa2* or *Δfaa2* strain was similar to that of the wild-type strain, indicating the existence of one pathway in cells. In contrast, the growth rate of the *Δfaa2/pxa2* strain decreased significantly (Fig. 8A). The plasmid containing the wild-type *pxa2* gene was delivered to the *Δfaa2/pxa2* strain, and cell growth was recovered to the same extent as that of the *Δfaa2* strain, indicating the presence of a functional Pxa1p-Pxa2p pathway (Fig. 8B). In contrast, the mutated *pxa2_Y726L_* or *pxa2_F731A_* gene only partially complemented the *Δfaa2/pxa2* strain, indicating the defective function of these two mutant proteins. Taken together, these results suggest that residues Y726 and F731 in the CT_2_ of Pxa2p are critical in protein-protein interaction and its transporter function.

### Two-hybrid analysis of the interactions between Pxa2_NBD and Pxa2_CT

**Figure 9:**
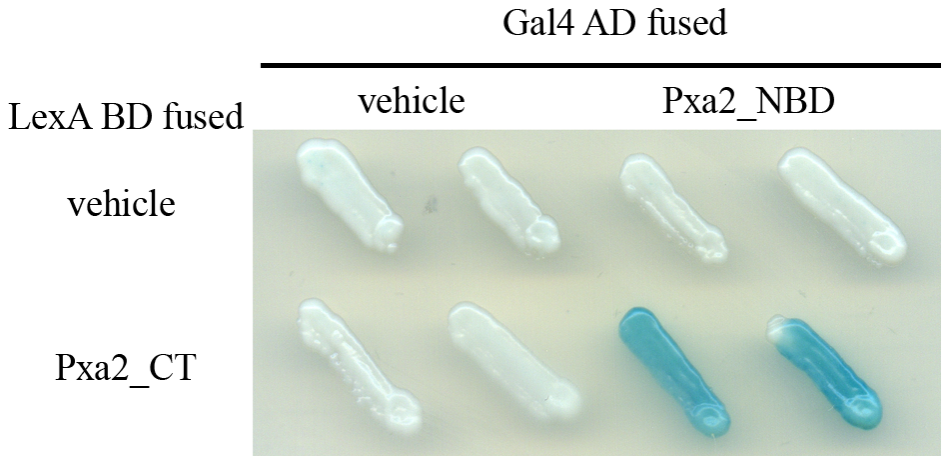
Two-hybrid analysis of interactions between Pxa2_NBD and Pxa2_CT. Pxa2_NBD was fused to Gal4 AD and Pxa2_CT was fused to Lex BD in yeast two-hybrid plasmids. Two-hybrid analysis was performed as described in Materials and Methods.

Finally, because the CT_2_ of Pxa2p is critical in altering the NBD domain to the interaction-competent state, it is possible that the CT of Pxa2p could interact with its NBD domain. To examine this possibility, we assayed the interaction between the NBD and the CT of Pxa2p by using yeast two-hybrid analysis. There indeed existed an interaction between the NBD and the CT of Pxa2p (Fig. 9). This result suggested that the CT may interact with the NBD in Pxa2p to form the interaction-competent structure.

## Discussion

In this study, we found that the CT of Pxa2p is required for the stable interaction of two NBDs in Pxa1p and Pxa2p. An interaction was not detected between their NBDs, as the CT of Pxa2p was deleted. Interestingly, the involvement of the CT of Pxa2p in the Pxa1p-Pxa2p interaction does not occur through direct contact with Pxa1_NBD, but in a Pxa2_NBD-dependent manner. The limited proteinase K digestion results of Pxa2p mutants defective in CT_2_ showed that the CT of Pxa2p stabilizes the structure of Pxa2p_NBD to that required for interaction with Pxa1p. The oleate plate functional assay clearly demonstrated that the CT_2_ of Pxa2p is required for transporter function, suggesting that the interactions identified here are functionally related. Finally, the proposition that the involvement of the CT of Pxa2p in maintaining its NBD structure was also corroborated by the interaction between the NBD and CT of Pxa2p in the yeast two-hybrid assay.

A number of studies have suggested that the C-terminal extension of ABC proteins may be associated with protein-protein interaction between two half ABC transporters. In human ALDP, a study using the yeast two-hybrid assay appeared to show that homo- and heterodimerization occur between C-terminal halves of ALDP, ALDRP, and PMP70 [4]. Another study showed that in MalK of *E. coli*, its regulatory C-terminal domains remained associated throughout the catalytic cycle, suggesting that the C-terminal domain is involved in stabilization of the MalK dimer [33]. In DrrA of *Streptomyces peucetius*, one study used the disulfide cross-linking assay to show that the LDEVFL motif in the extreme C terminus of DrrA is involved in DrrA-DrrB and DrrA-DrrA complex formation [15]. Similarly, in our study, we found that the CT of Pxa2p, but not Pxa1p, is required for Pxa1_NBD-CT/Pxa2_NBD-CT dimeric interaction. As clearly demonstrated, the interaction between Pxa1_NBD and Pxa2_NBD-CT may be directly mediated by their NBD domains with the help of the CT of Pxa2p. We could not, however, rule out the possibility that there exists a minor interaction between the CT of Pxa1p and that of Pxa2p; this idea will be addressed in further work.

An interesting finding in this report is that the involvement of the CT_2_ of Pxa2p in the Pxa1_NBD/Pxa2_NBD-CT interaction does not occur through direct contact with Pxa1_NBD (Fig. 5A). Instead, the CT_2_ of Pxa2p is required for maintaining the correct structure of Pxa2_NBD in order for it to interact with Pxa1_NBD. The genetic and biochemical results on the point mutations in the CT_2_ of Pxa2p support this suggestion (Figs. 6 and 8). The limited proteolysis assay of these point mutations indicated that new bands appeared on comparison of the peptide map of the wild-type protein and the CT_2_-mutated proteins, implying that the mutations located in the CT_2_ of Pxa2p indeed change the Pxa2_NBD-CT structure to a significant extent. Thus, we propose that the CT of Pxa2p may be structurally integrated into the interaction competency of the NBD in Pxa2p. In this model, the CT of Pxa2p should interact with the NBD of Pxa2p. To test this possibility, we analyzed the interaction between the CT and the NBD of Pxa2p by using a yeast two-hybrid assay. The results of this assay were in agreement with our proposed model (Fig. 9). Further analytical techniques are required to reveal the whole picture regarding the structural details of the integration of the CT into the complete NBD.

We also noticed that the CT_3_ of Pxa2p has an interesting property. When we compared the yeast two-hybrid results of Pxa1_NBD/Pxa2_NBD-CT_1+2_ and those of Pxa1_NBD/Pxa2_NBD-CT, it appeared that the protein-protein interaction was enhanced in Pxa1_NBD/Pxa2_NBD-CT_1+2_ (Fig. 3B). This enhancement was estimated as being up to 22% by β-galactosidase activity assay (data not showed). This result indicates that the CT_3_ of Pxa2p may have an inhibitory effect on the interaction between Pxa1_NBD and Pxa2_NBD-CT. Several reports have suggested that the C termini of ABC transporters are involved in regulating ATP binding and NBD interaction [34]–[36]. One of these biochemically well-characterized termini is the ATP-sensitive potassium channel (K_ATP_ channel), consisting of Kir6.2/SUR2. Four Kir6.2 proteins form the central pore in the membrane, each associated with a regulatory sulfonylurea receptor (SUR2) subunit, which contains TMD, NBD, and the C-terminal extension. Because the SUR2 gene mRNA is alternatively spliced in different tissues, there exist two protein isoforms, SUR2A and SUR2B, differing only in their C-terminal 42 amino acids. Compared with SUR2A, purified SUR2B has been shown to have lower ATPase activity and lower K_M_ for MgATP. Their different activities have been proven to result from an inhibitory effect of the C-terminal 42 amino acids of SUR2B. In future, it will be worthwhile to examine the deletion effect of the CT_3_ on its ATPase activity in order to characterize the regulatory role of the CT_3_ of Pxa2p.

The mutations Y726L and F731A in the CT_2_ of Pxa2p, shown in the present study to disrupt Pxa1_NBD/Pxa2_NBD interaction, correspond to the mutations W679R and L684P in the CT_2_ of human ALDP. From the records in the human X-ALD database (http://www.x-ald.nl/), we found that W679R was the first missense mutation discovered in exon 10 of the ALDP gene [37]. This mutation existed in sibling patients with adrenomyeloneuropathy. The patient and his sister, who was older by two years, had slow neurological progression with disturbances of balance and walking at about the age of 40–50 years, and the levels of very long-chain fatty acids in their blood were found to be elevated. An L684P mutation was recorded only in unpublished data by Dr. S.J.S. Steinberg. To date, the protein stability and biochemical properties of these two mutated proteins have not yet been described, and their pathological mechanisms are not clear. Because Pxa2p is the ortholog of ALDP, the mutation effect of Y726L and F731A in Pxa2p on its protein interaction with Pxa1p may provide a significant reference for ALDP protein with W679R and L684P mutations. Thus, the effects of the W679R and L684P mutations in ALDP on protein-protein interaction are worth determining in future. An accurate description of the mutation effect in ALDP may be useful for other pharmacological studies in order to discover drugs that enhance the interactions of mutated proteins and to provide improved treatment for ALD disease.

## Materials and Methods

### Yeast strains, culture medium, and antibodies

The yeast strains used in this study were *S. cerevisiae* BJ2168, EGY48 (pSH18-34), and W303.1A, which were kindly provided by Dr. S. C. Cheng (Institute of Molecular Biology, Academia Sinica, Taipei, Taiwan, Republic of China). Yeast cells were grown on liquid or solid YPD (1% yeast extract, 2% peptone, and 2% glucose with/without 2% agar) or minimal medium (0.7% yeast nitrogen base, 2% glucose or galactose, and appropriate amino acids). Anti-LexA BD and anti-Gal4 AD antibodies were purchased from Santa Cruz Biotechnology (Dallas, TX). Anti-HA and anti-His antibodies were purchased from GeneMark (Taiwan, ROC). Horseradish peroxidase (HRP)-conjugated secondary antibody was purchased from Millipore (Billerica, MA).

### Plasmid constructions and mutagenesis

The DNA fragments encoding the CT of Pxa1 and Pxa2 (Pxa1_NBD-CT, residues 500–870; Pxa1_NBD, residues 500–774; Pxa2_NBD-CT, residues 399–853; Pxa2_NBD-CT_1+2_, residues 399–773; Pxa2_NBD-CT_1_, residues 399–723; Pxa2_NBD, residues 399–677) were amplified from yeast genomic DNA by PCR reaction with appropriate primers. PCR products were cloned into pACT2 (with the Gal4 AD gene) and pEG202 (with the LexA BD gene) for a yeast two-hybrid assay or a pRS416-Gal and pRS414-Gal yeast expression vector for a pull-down assay. Pxa2_NBD-CT point mutations (Y726L and F731A) were individually introduced into pEG202-Pxa2-NBD-CT and pRS414-Pxa2-NBD-CT by PCR site-directed mutagenesis using “overlap extension” with Pfu polymerase and appropriate primers, as described [38]. Chimeric genes of Pxa2_NBD-CT_A1-CT2_, Pxa1_NBD-CT_A2-CT2+CT3_, and Pxa1_NBD-CT_A2-CT2_ were constructed in pEG202 by PCR splicing using overlap extension with appropriate primers. The accuracy of sequences of all cloned PCR products mentioned above was confirmed by automatic DNA sequencing.

### Two-hybrid assays

An EGY48 (pSH18-34) yeast reporter strain with plasmid-encoded LexA-inducible LacZ was co-transformed with the two-hybrid plasmids (pACT2/pEG202 or their derivatives containing a DNA fragment from Pxa1p or Pxa2p). The yeasts were then plated onto selected medium lacking uracil/histidine/leucine for plasmid keeping as described [39]. After incubation for 2–3 days, several colonies were picked out and streaked on the same medium again to make sure there was no contamination of undesirable cells lacking any plasmid. The yeast strains were then streaked onto an X-gal plate to measure β-galactosidase expression for determining protein-protein interaction.

### Cell disruption methods

#### Sonication

*Saccharomyces cerevisiae* cultures, grown as described above, were centrifuged at 12000 rpm for 10 min at 4°C and the cell pellet collected. Next, 10 ml Tris-buffered saline (TBS; 10 mM Tris buffer, pH 7.6 and 150 mM NaCl) was added to resuspend the 4 ml cell pellet. Cell suspension was then subjected to ultrasonic disruption at 30% energy on ice with 10 cycles of 5 s on and 15 s off. Cell debris was then removed by centrifugation at 12000 rpm for 15 min at 4°C. The supernatant was collected and stored on ice until use.

#### Glass bead

After resuspending 0.2 ml of the yeast cell pellet in 0.4 ml TBS, 1 g glass beads (Sigma, Ronkonkoma, NY) were added to each tube. Cells were then disrupted by vortexing vigorously for 20 min at 4°C. At the end of vortexing, the tube was centrifuged at 12000 rpm for 15 min at 4°C, and the supernatant (total lysate) was collected. The protein concentration of total lysate was determined by BCA reagent (Sigma).

### Western blot analysis

Western blot analysis followed the method described by Hsieh *et al*. [40] with minor modifications. Protein samples were separated by 12% SDS-polyacrylamide gel electrophoresis (SDS-PAGE) and then transferred onto polyvinylidene difluoride (PVDF) membranes (Millipore). After blotting, PVDF membranes were incubated in blocking buffer containing 5% non-fat dry milk in TBS buffer for 30 min at room temperature, followed by the addition of primary antibody and overnight incubation at 4°C. PVDF membranes were then washed three times in TBS for 10 min, followed by incubation in TBS with HRP-conjugated secondary antibody (1∶5000) for at least 2 h at room temperature. Protein signals were detected after the adding of Immobilon Western Chemiluminescent HRP Substrate (Millipore). Signals were quantitated by using GeneTools software (SynGene, Frederick, MD) when required.

### Ni-NTA affinity chromatography

Cell extracts were prepared by sonication from yeast strain BJ2168 co-expressing Pxa2_NBD-CT-His/Pxa1_NBD-HA, Pxa2_NBD-CT_1+2_-His/Pxa1_NBD-HA, Pxa2_NBD-CT_1_-His/Pxa1_NBD-HA, or Pxa2_NBD-His/Pxa1_NBD-HA. One milliliter of Ni-NTA resin (Bio-Rad, Hercules, CA) was pre-equilibrated with a 15× volume of equilibration buffer (10 mM Tris buffer, pH 7.6, and 150 mM NaCl) at 4°C and then loaded onto a 10×1 cm (length×diameter) column. The yeast extracts were passed through Ni-NTA columns with a flow rate of 0.5 ml/min at 4°C. After sequential washings with a 20× volume of equilibration buffer containing 25 mM imidazole at 4°C, the bound proteins were eluted with the same buffer containing 250 mM imidazole at 4°C and collected at 1 ml/fraction. Proteins collected in each fraction were precipitated with trichloroacetic acid at a final concentration of 12% (w/v) and analyzed by SDS-PAGE and Western blot.

### β-Galactosidase assay

The β-galactosidase assay was performed by using the Yeast β-Galactosidase Assay Kit (Thermo), following the manufacturer's instructions. The OD_660_ of each yeast culture was adjusted to 1 by using fresh medium, and 70 µl of culture was dropped into the 96-well plate. Lysis buffer (70 µl) was added to each well and incubated for 30 min at 30°C. A stop solution (56 µl) was then added to each well to stop the reaction. The 96-well plate was placed in an ELISA reader to measure the absorbance of each well at 420 nm. To calculate β-galactosidase activity, we used the equation provided by the manufacturer: 1000×A420/*t*×*V*×OD_660_. In this equation, *t* was 30 min and *V* was 196 µl.

### Limited proteolysis by proteinase K

*Saccharomyces cerevisiae* BJ2168 expressing Pxa2_NBD-CT, Pxa2_NBD-CT_Y726L_, or Pxa2_NBD-CT_F731A_ proteins was disrupted by the glass bead method. The same protein level for each lysate was digested with serial dilution of proteinase K (MDBio, Taiwan, ROC) in TBS buffer for 10 min at 25°C. At the end of the reaction, PMSF was added at 1 mM to stop the reaction. The sample buffer for SDS-PAGE was then added to each tube. All samples were boiled for 10 min at 100°C. The samples were analyzed by SDS-PAGE and Western blot.

### Oleate plate assay

The yeast strains used in this assay were *S. cerevisiae* W303.1A. All mutant strains (*Δpxa2, Δfaa2, Δpex19, and Δfaa2/Δpxa2*) were constructed with a two-step plasmid integration-excision protocol [41]. The plasmid-encoded Pxa2p wild-type and mutant proteins were full-length proteins with a complete transmembrane region, and their genes were constructed in the pRS414 vector with the cognate promotor sequence upstream of the transcription start site. The oleate plate is composed of 0.7% yeast nitrogen base, 0.5% potassium phosphate buffer (pH 6.0), essential amino acids, 1.6% bacto-agar, and a carbon source (0.4% oleic acid dissolved in 2% Tween40 or 2% glucose). The assay was started by preincubating yeast strains in minimal medium supplemented with essential amino acids and 2% glucose. As the OD_600_ of each yeast culture was near 5, the same amount of yeast cells (1 OD_600_×1 ml) was pelleted and resuspended in 1 ml sterile water and then allowed it to stand for 30 min. This step was repeated three times for complete glucose depletion in cells. Five-fold serial dilutions using sterile water were then performed. Cells from each dilution (3 µl) were dropped onto the oleate plate directly, followed by incubation at 30°C for 5–7 days.
